# The detection of foodborne bacteria on beef: the application of the electronic nose

**DOI:** 10.1186/2193-1801-2-687

**Published:** 2013-12-23

**Authors:** Soad A Abdallah, Laila A Al-Shatti, Ali F Alhajraf, Noura Al-Hammad, Bashayer Al-Awadi

**Affiliations:** General Science Unit, College of Nursing, Public Authority for Applied Education & Training (PAAET), leave from Women’s College for Arts, Science & Education, Botany Department, Ain Shams University, Al-Shuwaikh B, PO Box 64923, Kuwait City, 70466 Kuwait. On tenured Cairo, Egypt; Biomedical Sciences Department, College of Nursing, PAAET, Kragujevac, Kuwait City, Kuwait

**Keywords:** Food, Pathogens, Rapid detection, Electronic nose

## Abstract

This study aims to investigate the application of a fast electronic nose system (Cyranose 320) for detecting foodborne bacteria. The system proved to be very efficient in detecting microbes in beef and sausage samples. In the first part of the study, the total viable counts (TVC) from fresh and frozen beef samples were determined using the standard microbiological method and by the application of the electronic nose. The second part applied the electronic nose to beef before and after contamination with different bacterial pathogens separately: *E. coli* O157: H7, *Salmonellatyphimurium* 857, *Staphylococcus aureus* 29213 and *Pseudomonas aeruginosa* 27853. The results revealed that the Cyranose 320 can detect the TVC in different beef and sausage samples and quantify the volatile organic compounds produced at concentrations from 50 ppb to > 350 ppb. The concentrations of gases collected from the samples before and after separate contamination with these pathogenic bacteria were highly significantly correlated (P < 0.005). From this study one can conclude that the electronic nose system is a rapid way for detecting volatile organic compounds produced by foodborne bacteria that contaminate beef.

## Background

Foodborne pathogens such as bacteria or toxins, viruses or parasites may lead to human disease when contaminated food is eaten. The source of contamination may vary but harmful bacteria are mostly responsible for causing gastrointestinal infections (Scallan et al. 2011). The sources could be the animal, the environment or contamination during food processing (McNamara 1998; Slutsker et al. 1998). The main source of meat contamination is animal feces especially during processing at the slaughterhouse (Kudva et al. 1998). Food animals and poultry are the most important reservoirs for many foodborne pathogens (Biswas et al. 2008). Foodborne illnesses associated with meat are caused mostly by certain types of bacteria namely *Bacillus cereus*, *Campylobacter jejuni*, *Clostridium botulinum*, *Clostridium perfringens*, *Escherichia coli* O157:H7, *Listeria monocytogenes*, *Salmonella* sp., *Staphylococcus aureus*, *Pseudomonas aeruginosa* (Atlas and *Yersinia enterocolitica*^1998^; FSIS 1998; Beran et al. 1991; Doyle et al. 1997).

Existing methods for preventing microbial diseases depend on controlling different types of pathogenic bacteria by food safety management and through medical and environmental observation (Meng and Doyle 1998). Classical methods for the cultivation, isolation and identification of bacteria usually comprise morphological assessment in addition to determining the ability of microorganisms to grow in different media under different growth conditions (Ivnitski et al. 2000).

The polymerase chain reaction (PCR) is mainly used in research in food microbiology for the identification of bacterial genus and species due to its high efficacy and accuracy. Although PCR is an advanced technology, inhibitors that occur in foods or the culture media could affect the reaction. However, this technology cannot differentiate between active cells and inactive dead cells so could lead to false results and thus fail to reflect bacterial numbers accurately (Mandal et al. 2011).

Instruments used in the food industry can help to detect pathogens. One example, the electronic nose (e-nose), is an electronic instrument that is able to imitate the human ability to detect odor. The Cyranose 320, used in this work contains a group of up to 32 sensors, each one made of a complex of conductive carbon black blended with a non-conductive polymer with each sensor being able to differentiate between different types of gas, whether pure or mixed. Using the e-nose allows an immediate decision regarding the quality of the sample without leaving the sampling location (Hobbs 2003; Mandal et al. 2011). Winquist et al. (1993) showed that the electronic nose could be used within the medical environment and in the food industry where it could discriminate between types of ground beef and assess quality during storage. Many studies (Falasconi et al. 2012; Sayeed and Shameen 2011; Berna 2010; Dutta et al. 2002; Rossi et al. 1995; Holmberg et al. 1998; Gardner et al. 1998) have used a particular e-nose instrument - First Generation E-Nose, Second Generation E-Nose and Third generation E-Nose especially Cyranose 320- to classify bacteria and to discriminate between different species. This instrument has also been used to detect the presence of *E. coli* in samples (Powell et al. 2002) and to detect the production of volatile compounds relating to chicken storage time and temperature (Boothe and Arnold 2002).

The aim of this study is to compare detection methods for foodborne bacteria such as *Salmonella sp.*, *Staphylococcus aureus*, *Pseudomonas aeruginosa* and *E. coli* O157:H7 from different types of beef using the electronic nose and the standard methods.

## Results

The present study is divided into two parts: firstly, a cross validation test for determining the total viable count (TVC) on samples of fresh beef (cut or minced), frozen beef (cut or minced) and sausages (fresh or frozen) on five consecutive days using both the routine method (TVC) and the e-nose and secondly, using the e-nose to detect gases from a range of bacteria contaminating similar beef and sausage samples.

Table 1 shows the total viable counts (TVC): for fresh cut beef samples, it increased up to day 2 then decreased by day 4 then increased to the highest level on day 5, ranging from 1.22 × 10^3^ to 2.42 × 10^5^ CFU/ml. while fresh beef minced samples gave lower levels of TVC which decreased by day 2 then increased on days 3, 4 and 5, ranging from 4.44 × 10^2^ to 6.10 × 10^4^ CFU/ml. For fresh sausage samples the TVC was zero on day 1 and then increased to reach 2.43 × 10^5^ CFU/ml on day 5. The frozen cut beef showed the highest TVC levels on day 1, decreased by days 2 and 3 then increased on day 4 before decreasing sharply on day 5, ranging from 1.11 × 10^2^ to 6.62 × 10^4^ CFU/ml. Frozen minced beef started with the highest TVC level on day one (1.37 × 10^5^ CFU/ml) which then fluctuated during the next 4 days to reach its lowest level on day 5 (1.11 × 10^2^ CFU/ml). The TVC of the frozen sausage samples fluctuated, reaching its highest value on day 4 (3.71 × 10^5^ CFU/ml) then decreasing to its lowest value on day 5 (2.22 × 10^2^ CFU/ml).

**Table 1:** **Total viable count (TVC) CFU/ml of beef and sausage samples**

Samples	Fresh cut	Fresh minced	Fresh sausage	Frozen cut	Frozen minced	Frozen sausage
Day 1	1.33 × 10^3^	1.00 × 10^3^	1.00 × 10^1^	6.62 × 10^4^	1.37 × 10^5^	2.24 × 10^4^
Day 2	1.70 × 10^5^	4.44 × 10^2^	5.11 × 10^3^	5.33 × 10^3^	6.67 × 10^2^	1.95 × 10^5^
Day 3	1.53 × 10^5^	5.41 × 10^4^	1.11 × 10^3^	4.33 × 10^3^	3.67 × 10^3^	8.33 × 10^2^
Day 4	1.22 × 10^3^	1.03 × 10^4^	5.67 × 10^4^	6.39 × 10^4^	1.56 × 10^3^	3.71 × 10^5^
Day 5	2.42 × 10^5^	6.10 × 10^4^	2.43 × 10^5^	1.11 × 10^2^	1.11 × 10^2^	2.22 × 10^2^

According to a statistical analysis using the paired *t*-test, there was no significant difference between the TVC values from fresh cut and fresh minced beef samples (P > 0.05). The same results were obtained between fresh cut beef and fresh sausage, and between fresh minced beef and fresh sausage (P > 0.05) (Table 1).

Correlation matrix analysis is about observing the interaction of various beef samples’ TVC. For instance, correlation is computed into what is known as the correlation coefficient, which ranges between -1 and +1. Perfect positive correlation (a correlation coefficient of +1) implies that as TVC of one beef sample moves, either up or down, the other sample’s TVS will move in the same direction. Alternatively, perfect negative correlation means that if TVC of a beef sample’s moves in either direction the TVC of the other sample that is perfectly negatively correlated will move in the opposite direction. If the correlation is 0, the sample’s TVCs are said to have no correlation.

The correlation matrix using the TVC data from the different types of beef examined in the present study (Table 2), showed that there was a very strong negative correlation between frozen and fresh cut beef (-0.966), a positive moderate correlation between fresh cut and fresh minced beef (0.681) and between fresh cut and fresh sausage samples (0.560), and between fresh sausage and fresh minced beef (0.621). However, negative weak correlations occurred between frozen sausage and fresh sausage samples (-0.205), between frozen minced beef and fresh sausage (-0.341) and between frozen minced with fresh sausage samples (-0.330). According to the P value of all correlations were not significant (P > 0.05) except the correlation between the fresh and frozen cut samples were highly significant (P < 0.01).

**Table 2:** **Correlation matrix between TVC values from all beef and sausage samples**

Samples	Fresh minced	Fresh sausage	Fresh cut	Frozen sausage	Frozen minced
Fresh sausage	0.621				
Fresh cut	0.681	0.560			
Frozen sausage	-0.564	-0.205	-0.470		
Frozen minced	-0.453	-0.341	-0.587	-0.330	
Frozen cut	-0.640	-0.344	-0.966*	0.440	0.631

*indicates its P value <0.01.

On the basis of these findings, the frozen minced beef and frozen sausage samples were excluded from the e-nose application because frozen samples gave less TVC when using the routine method (TVC).

**Figure 1:**
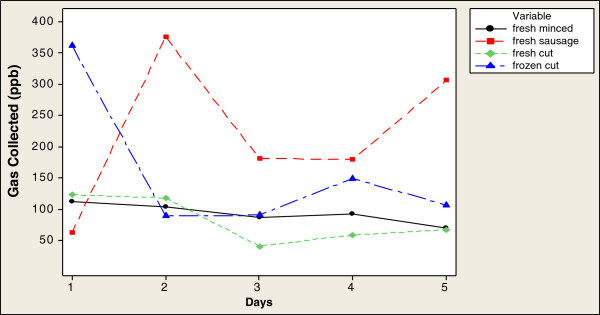
**Time series plot of gas concentrations detected by e-nose over 2 h.**

**Figure 2:**
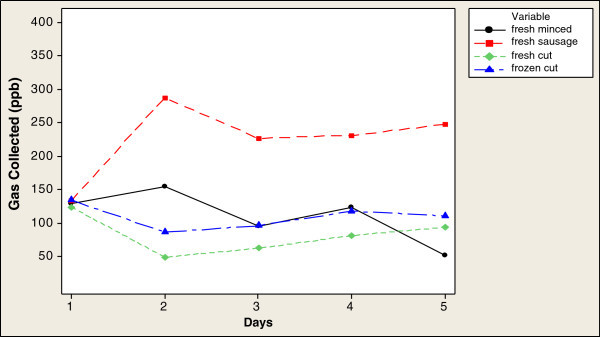
**Time series plot of gas concentrations detected by e-nose over 4 h.**

From the e-nose data, gases produced by the viable bacteria gave higher concentration values after 2 h than after 4 h (Figures 1 and 2). Fresh sausage samples produced the highest concentration of gases, exceeding 350 ppb on day 2 (Figure 1), decreasing to < 200 ppb on day 3, while frozen cut beef samples also showed a high gas concentration, exceeding 350 ppb on day 1, followed by fresh cut beef and fresh minced beef.

After 4 h, all samples showed lower concentrations of gases than after 2 h (Figure 2). Fresh sausage showed the same pattern as after 2 h: it showed the highest concentration of gas collected by the e-nose especially on day 2 when it exceeded 250 ppb, decreasing on days 3 and 4 to fall below 250 ppb. The concentration of gas detected in the fresh minced beef reached 150 ppb on day 2 then decreased to below 100 ppb on day 3 and above 100 ppb on day 4. Fresh cut beef samples showed the lowest gas concentration at below 50 ppb on day 2 (Figure 2) these changes in the gas collected might be attributed to the beef sample type.

After applying the paired *t*-test to compare the gas concentrations on day 1 with day 5, no significant differences were detected (P > 0.05) for all types of beef sample (fresh cut, fresh minced and frozen cut). In contrast, a highly significant difference was found between values for fresh sausage on day 1 and day 5, whether after 2 or 4 h (P = 0.000 and 0.009 respectively) (Table 3).

**Table 3:** **Comparison of gas concentrations (ppb) on day 1 and day 5 for fresh cut and minced beef, fresh sausage and frozen cut beef after 2 and 4 h**

Samples	Two hours			Four hours		
	Mean	SD	P value	Mean	SD	P value
Fresh cut	56.0	22.7	0.997	30.2	20.18	0.986
Fresh minced	42.4	40.8	0.957	76.8	28.4	0.994
Fresh sausage	-244	20.91	0.000**	-114	65.8	0.009**
Frozen cut	253	47	1.000	23.8	22.9	0.960

**indicates P <0.01.

The second part of this study was to apply the e-nose to fresh beef samples (cut, minced and sausage) before and after contamination with the different types of bacterial pathogens *E. coli* O157: H7, *Salmonella typhimurium* 857, *Staphylococcus aureus* 29213 and *Pseudomonas aeruginosa* 27853, after 2 and 4 h.

**Figure 3:**
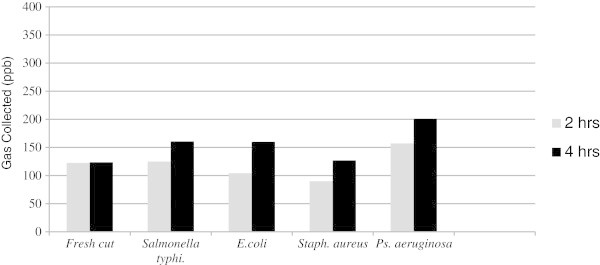
**Concentration of gases (ppb) collected by e-nose from fresh cut beef before and after bacterial contamination.**

Figure 3 shows the concentrations of gas collected after 2 and 4 h using the e-nose from the uncontaminated fresh cut samples and those contaminated separately by the above-mentioned pathogens. The data shows that the concentration of gases collected from the fresh cut beef after 4 h was more than that collected after 2 h for all contaminated samples. While the uncontaminated control sample showed the same concentration of gases after 2 and 4 h (>100 ppb), *Pseudomonas aeruginosa* produced the highest concentration of gases collected after 4 h reaching 200 ppb, followed by *E. coli* and *Salmonella typhimurium* which produced gas concentrations above 150 ppb.

**Figure 4:**
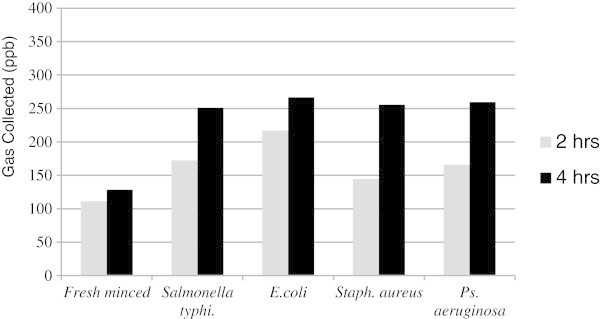
**Concentration of gases (ppb) collected by e-nose from fresh minced beef before and after bacterial contamination.**

For fresh minced beef, the data revealed that the concentration of gases collected from samples exposed to pathogenic bacteria showed differences in value after 2 h. *E. coli* showed the highest concentration of gases collected, exceeding 200 ppb, followed by samples contaminated with *Salmonella typhimurium* (> 150 ppb), *Pseudomonas aeruginosa* and *staphylococcus aureus* (Figure 4). The uncontaminated fresh minced beef samples produced a gas concentration of 100 ppb after 2 h.

**Figure 5:**
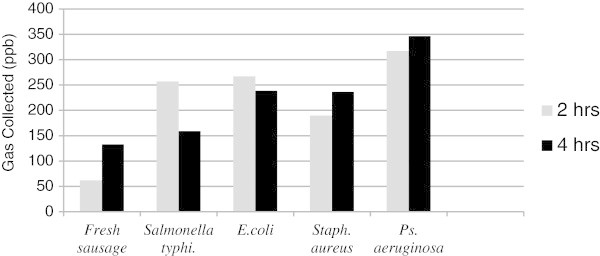
**Concentration of gases (ppb) collected by e-nose from fresh sausage before and after bacterial contamination.**

For the fresh sausage sample, the concentration of gases produced reached 350 ppb from *Pseudomonas aeruginosa* after 4 h and above 300 ppb after 2 h. *E. coli* produced concentrations above 250 ppb after 2 h but below 250 after 4 h. However, *Salmonella typhimurium* produced gas concentrations of 250 ppb after 2 h which decreased to 150 ppb after 4 h. *Staphylococcus aureus* produced concentrations above 200 ppb after 4 h and below 200 ppb after 2 h. The lowest concentration of gases collected by the e-nose came from the uncontaminated fresh sausage (Figure 5).

Using the paired *t*-test, comparing the concentration of gases collected after 2 h on days 1 and 5 showed a significant difference for fresh minced beef contaminated with *E. coli* (P < 0.05) and highly significant differences for fresh sausage contaminated by *E. coli* (P < 0.005). Another significant difference occurred between the concentrations of gases produced on days 1 and 5 by *Salmonella typhimurium* in the fresh sausage samples (P < 0.05). Highly significant differences were found between the concentrations of gases collected from fresh sausage exposed to *Staphylococcus aureus* and *Pseudomonas aeruginosa* (P < 0.005). Significant differences also occurred between the concentrations of gases collected from fresh cut beef samples exposed to *Staphylococcus aureus* and *Pseudomonas aeruginosa* (P < 0.05) (Table 4). When the paired *t*-test was also applied to the gas concentrations after four hours collected on days 1 and 5 (Table 5), it showed highly significant differences between those from minced beef exposed to four different types of pathogenic bacteria separately (P ≤ 0.005). Another highly significant difference was found with fresh cut beef and fresh sausage exposed to *Pseudomonas aeruginosa* (P < 0.005). A significant difference was also revealed between the amounts of gases collected from fresh cut beef and fresh sausage after contamination with *E. coli* (P < 0.05).

**Table 4:** **Comparison of mean gas concentrations (ppb) on days 1 and 5 from fresh cut and minced beef and fresh sausage samples after 2 h**

Samples	***E. coli***			***Salmonella typhimurium***			***Staphylococcus aureus***			***Pseudomonas aeruginosa***		
	Mean	SD	P value	Mean	SD	P value	Mean	SD	P value	Mean	SD	P value
Fresh cut	18.6	23.5	0.151	-2.2	24.3	0.850	32.8	22.9	0.033*	-34.4	20.96	0.021*
Fresh minced	-106	65.0	0.022*	-61.2	50.9	0.055	-33.4	29.9	0.067	-54.8	46.8	0.059
Fresh sausage	-205	67.9	0.003**	-195.2	136.5	0.033*	-127.8	20.86	0.000**	-255.2	26.2	0.000**

**indicates P < 0.01, *P < 0.05.

**Table 5:** **Comparison of mean gas concentrations (ppb) on days 1 and 5 from fresh cut and minced beef and fresh sausage samples after 4 h**

Samples	***E coli***			***Salmonella***			***Staphylococcus aureus***			***Pseudomonas aeruginosa***		
	Mean	SD	P value	Mean	SD	P value	Mean	SD	P value	Mean	SD	P value
Fresh cut	-36.2	24.2	0.029*	-37.0	33.2	0.067	-3.4	25.4	0.780	-77.40	20.48	0.001**
Fresh minced	-138	55.2	0.005**	-122.4	44.5	0.004**	-127.2	47.6	0.004**	-131.2	33.6	0.001**
Fresh sausage	-105	78.1	0.039*	-26.0	85.9	0.536	-103.2	87.0	0.057	-213.2	67.1	0.002**

**indicates P < 0.01, *P < 0.05.

Regarding the potentiometric analysis of the sodium nitrate (NaNO_3_) content in meat, the results were 19.90, 23.28, and 67.50 ppm in fresh cut beef, fresh minced beef and fresh sausage, respectively.

## Discussion

Quality assurance methods in the food industry need to be specific and selective for microbiological examinations. Microbiological processes occurring during the storage of raw meat lead to wastage which threatens meat industry economics and causes a challenging problem form eat businesses. Quantitative assessments of beef contamination can be made by a routine total viable count (TVC) and other tests which measure the number of bacteria (TVC) in a sample that can survive in the conditions on the surface of raw meat or in processed meat, can be harvested by the sampling procedure used and can grow in the presence of air on an agar plate. These bacteria originate both from animals and from the slaughterhouse or meat processing environment. As the TVC includes organisms contaminating the meat, it will also give an indication of its keeping quality. The disadvantages of these routine methods are that they are laborious, costly and time-consuming but these can be overcome by a rapid method that gives instant or real-time results. Rapid methods not only provide the early detection and enumeration of microorganisms but can also characterize these isolates (Naravaneni and Jamil 2005). To determine the extent of spoilage in raw meat, the standard method used to analyze the total viable count of bacteria requires a 1–2 day incubation period to form a bacterial colony on agar plates. Although the literature shows that bacterial growth in meat samples has been widely studied, there is still a need for research on methods to correlate the number of bacteria with shelf-life determination (Panigrahi et al. 2006).

In the present study, the results support using the electronic nose for bacteriological examinations instead of the alternative routine methods. An electronic nose and other measurements were used on beef samples including minced meat and sausages over a period of five days. To use the electronic nose system, the routine bacteriological method was applied as a reference method. The results showed that the highest level of bacterial counts in freshly cut meat occurred on the fifth day whereas in frozen samples, it was the fourth day.

The e-nose system can easily distinguish between spoiled and fresh meat. The positive result obtained indicated that this system can be effectively used to rapidly detect contamination.

It is important to note that the purpose of testing against the process criteria that have been set out for raw and certain processed meat is not to assess their fitness for human consumption but to provide an indication of performance and control of the slaughtering, dressing and production processes at the time of sampling and so must be used accordingly. If the criteria are not met, corrective action to improve future production must be initiated but there is no requirement to remove products from the market.

The experiments using the Cyranose e-nose have shown that viable bacteria can be detected in the contaminated samples. From our results, the e-nose has the potential to be used as a tool for the rapid detection of contamination, agreeing with the findings of Ding et al. (2010). The Cyranose 320 equipment has some advantages such as its ability to work with turbid media, high sensitivity and small size (Ivnitski et al. 2000). This electronic nose also has the advantages of short preparation time, being inexpensive to run, safe to use and efficient at volatile detection. Moreover, these e-noses are more sensitive to small bacterial cell numbers, have specificity towards different species of foodborne bacteria in addition to providing results in or near actual time without pre-enrichment. The most important challenge for screening a food sample is achieving the requirements for high sensitivity and a rapid time for effective analysis.

To assure human health, pathogenic microorganisms in meat products such as *Salmonella* need to be detected early. The pathogenic strains used in this study were detected by an electronic nose system designed to detect spoiled and unspoiled meat based on contamination. This agrees with previous studies which stated that the electronic nose could detect pathogenic organisms in beef (Balasubramanian et al. 2005; Kress-Rogers 1996; Vernat-Rossi et al. 1996). A study by Siegmund and Pfannhauser (1999) revealed that the electronic nose can detect *Salmonella typhimurium* at a contamination level of 0.7–2.6 log_10_CFU/g.

As mentioned in the results section, the e-nose detects the volatile organic compounds produced from sausage samples. Spices, which play a significant role in processed meat products, can be contaminated with a microbial load depending on the processing method, particle size, the variety and moisture content (Akgul 1993). The microbial load of the spice mixture increased that of the sausage samples so that the concentration of gas was higher than that from the beef samples. An instrument to detect sensory properties’ reproducibly is needed and could be an alternative to a sensory panel in the food industry (Haugen and Kvaal 1998).

Sodium nitrate can be used to extend food storage time without changing the food’s color, taste, odor and nutritional value and is used as a preservative in meat curing preparations and meat products at not more than 500 ppm (Branen et al. 1990; Dich et al. 1996; Hsu et al. 2009; Food and Drug Administration 2013). The concentration of gas collected by the e-nose is inversely proportional to the nitrate content of the meat due to its antimicrobial activity, this in harmony with previous studies (Sundberg et al. 2013). Unfortunately, in the present study, nitrate readings were totally different. This might be attributed to several different causes. The unprocessed meat showed a trend for higher microbial activity over4h compared with 2 h possibly because of the lack of antimicrobial additives but the processed meat showed the same trend. However, the differences between each bacterial species after 2 and 4 h decreased in the processed meat due to the presence of nitrate additives reducing microbial activity. Exceptionally, in *S. typhimurium* and *E. coli* on processed meats, the microbial activity decreased after 4 h compared with 2 h proving that NaNO_3_ is an effective microbial inhibitor towards *S. typhimurium* and *E. coli*.

## Conclusions

The current study suggests that the electronic nose can be used to detect meat contamination with the application of statistical analysis. It can easily run tests to check food products for spoilage, so it would be fast and effective in the food industry. However, there still is a need to ensure its reliability by conducting more research that aims to improve and assure the validity of the results. Gas sensing technologies can be used efficiently to help control food quality. However, this method does not replace reference methods such as using sensory panels because it needs standardization to become the equivalent of other validated reference methods. If the sample handling problems and other instrument performance issues of such a technique can be solved, the application of this instrument in the food industry would be very promising for understanding and assuring food contamination.

## Methods

### Sample collection

Six different types of beef samples were collected from a local market: cut fresh beef, minced fresh beef, cut frozen beef, minced frozen beef, processed fresh sausage and processed frozen sausage. All beef and sausage samples were cut into pieces weighing 10 g ±1, placed immediately in plastic bags and kept in are refrigerator at 4°C.

### Microbiological enumeration for contaminated beef samples

A 10 g sample was taken from the refrigerator, placed in 100 ml peptone water (Oxoid) (Thermo Scientific, UK) and left for two minutes (Sneath et al. 1986). Dilutions were prepared using the same diluents. Three dilutions were selected (10^3^, 10^5^ and 10^8^) and plated on Plate Count Agar (Oxoid). The plates were incubated at 30°C for 24-48 hrs. By enumerating the colonies present, the total viable counts (TVC) were obtained and calculated aslog_10_ colony forming units (CFU)/ml of the sample. This procedure was repeated every day for 10 days. The microbiological enumeration of the six types of beef and sausage samples under investigation was performed separately as mentioned above.

### Sample preparation for the e-nose

A 20 g beef sample was taken from the refrigerator and put into a 100 ml glass bottle and sealed with Parafilm® and held at room temperature (22°C ± 2°C) for two hours and four hours. After 2 h the tube that is connected to the Cyranose 320 was penetrated the parafilm® and dipped in the bottle without touching the liquid media. The Cyranose start button is pressed and its monitor started to detect the gas collected, within few seconds the reading is settled and taken. The instrument is re-set to take the second reading and so on. The same procedure is repeated after 4 h. Duplicate samples from each beef type were used. Measurements by the e-nose were performed after 2 and 4 h each day on 5 consecutive days for each duplicate and each sample (Nychas et al. 2008).

### Contaminated sample preparation

*E. coli* O157: H7, *Salmonellatyphimurium* 857, *Pseudomonas aeruginosa* 27853 and *Staphylococcus aureus* 29213 (Obtained from Faculty of Medicine, Kuwait University, Kuwait) were grown separately overnight at 37°C in a 125 ml flask to be used to inoculate the beef samples (Feng and Weagant 2002). The optical density of this culture was adjusted to give a concentration of about 100 colony forming unit per ml (CFU/ml). Beef and sausage samples (10 g) were placed in 250 ml glass bottles with the 100 ml bacterial cultures and sealed with Parafilm® to make it air-tight and incubated at room temperature 22°C. After intervals of 2 and 4 hours the gas produced by the bacteria was collected for measurement by the e-nose.

### Electronic nose application

The portable electronic nose, the Cyranose-320 (Model XP-329 III R, New Cosmos Electric Co. Ltd. Japan), can be used for detecting odors in a variety of industrial environments. By imprinting an odor print on its 32-sensor nose chip, arranged as an array, the e-nose can be used for non-invasive medical diagnostics, identification of hazardous materials, detection of food spoilage and many other applications. The sensors within the electronic nose are composed of a complex material consisting of conductive carbon black mixed with a non-conductive polymer. When the sensors are exposed to a gas, the polymer absorbs the gas and swells, during which the distance between the conductive carbon particles increases and thus also increases the resistance of the sensor material. This change in resistance is transmitted to a computer with the pattern of change in the sensor array being used to detect the gas.

### Potentiometric nitrate analysis

Fifty grams of meat sample were soaked separately in 250 ml peptone water (Oxoid) broth for 4 h. The nitrate content of the solution was measured by using a pH-meter (Hanna HI 2550 pH/ORP, Woonsocket, RI, USA) with a nitrate combination electrode (Hanna HI 4113).

### Statistical analysis

Statistical analysis was performed using the Minitab statistical package version 16 (Minitab Inc. 2010, State College, PA, USA). The paired *t*-test, time series and correlation matrix functions were applied to reveal any significant correlations and differences when using the Cyranose 320 using a P value < 0.005 (Daniel 2005).
